# Continuum of Care in a Maternal, Newborn and Child Health Program in Ghana: Low Completion Rate and Multiple Obstacle Factors

**DOI:** 10.1371/journal.pone.0142849

**Published:** 2015-12-09

**Authors:** Francis Yeji, Akira Shibanuma, Abraham Oduro, Cornelius Debpuur, Kimiyo Kikuchi, Seth Owusu-Agei, Margaret Gyapong, Sumiyo Okawa, Evelyn Ansah, Gloria Quansah Asare, Keiko Nanishi, John Williams, Sheila Addei, Charlotte Tawiah, Junko Yasuoka, Yeetey Enuameh, Evelyn Sakeah, Peter Wontuo, Masamine Jimba, Abraham Hodgson, Ghana EMBRACE Implementation Research Project Team

**Affiliations:** 1 Navrongo Health Research Centre, P.O. Box 114, Navrongo, Upper-East, Ghana; 2 Department of Community and Global Health, Graduate School of Medicine, University of Tokyo, 7-3-1 Hongo, Bunkyo-ku, Tokyo 113–0033, Japan; 3 Kintampo Health Research Centre, P.O. Box 200, Kintampo, Brong-Ahafo, Ghana; 4 Dodowa Health Research Centre, P.O. Box DD1, Dodowa, Greater Accra, Ghana; 5 Research and Development Division, Ghana Health Service, Accra MB 190, Ghana; 6 Ghana Health Service Headquarters, Accra MB 190, Ghana; Vanderbilt University, UNITED STATES

## Abstract

**Background:**

Slow progress has been made in achieving the Millennium Development Goals 4 and 5 in Ghana. Ensuring continuum of care (at least four antenatal visits; skilled birth attendance; postnatal care within 48 hours, at two weeks, and six weeks) for mother and newborn is crucial in helping Ghana achieve these goals and beyond. This study examined the levels and factors associated with continuum of care (CoC) completion among Ghanaian women aged 15–49.

**Methods:**

A retrospective cross-sectional survey was conducted among women who experienced live births between January 2011 and April 2013 in three regions of Ghana. In a two-stage random sampling method, 1,500 women with infants were selected and interviewed about maternal and newborn service usage in line with CoC. Multiple logistic regression models were used to assess factors associated with CoC completion.

**Results:**

Only 8.0% had CoC completion; the greatest gap and contributor to the low CoC was detected between delivery and postnatal care within 48 hours postpartum. About 95% of women had a minimum of four antenatal visits and postnatal care at six weeks postpartum. A total of 75% had skilled assisted delivery and 25% received postnatal care within 48 hours. Factors associated with CoC completion at 95% CI were geographical location (OR = 0.35, CI 0.13–0.39), marital status (OR = 0.45; CI 0.22–0.95), education (OR = 2.71; CI 1.11–6.57), transportation (OR = 1.97; CI 1.07–3.62), and beliefs about childhood illnesses (OR = 0.34; CI0.21–0.61).

**Conclusion:**

The continuum of care completion rate is low in the study site. Efforts should focus on increasing postnatal care within 48 hours and overcoming the known obstacles to increasing the continuum of care completion rate.

## Introduction

Although many efforts have been made over the past decade, most Sub-Saharan African (SSA) countries have difficulties in achieving the Millennium Development Goals (MDGs) 4 and 5 [1,2]. Annually, 2.9 million newborns and 265,000 mothers die due to pregnancy-related, and childbirth complications worldwide and SSA accounts for over half of these deaths [3]

To improve maternal, newborn, and child health (MNCH), the World Health Organization (WHO) and other organizations, over the past decade, have been advocating for continuum of care (CoC). It can provide as a key package of programs for MNCH, and can show a pathway to help reduce maternal and neonatal burden and deaths [4–7]. A potent CoC links crucial MNCH packages across the pregnancy, delivery, and postpartum stages. The benefits of CoC are that each stage builds on the success of the previous stage. For instance, antenatal care (ANC) visits to a health-care practitioner can prevent problems during pregnancy and increased the possibility of the mother receiving the appropriate care at birth [8]. Skilled care before, during, and immediately after birth reduces the risk of death or disability for both the mother and baby [9]. A lack of appropriate care at any levels of CoC is associated with poor MNCH outcomes [10–13]. This study focused on examining to what extent a woman and baby completed CoC across the pregnancy, delivery, and postpartum stages and identifying the gaps and barriers to CoC completion in Ghana.

### The Concept of CoC in MNCH

The concept of CoC first came to light in the 1970s in relation to the integration of research and practice for the provision of a CoC for older people [13]. For CoC in MNCH, time and place are the two dimensions that focus on its provision. The time dimension addresses the importance of linkages among the packages of MNCH service delivery at different stages during the pregnancy, childbirth, and postpartum periods. The place dimension links the various levels of care at home, community, and health facilities. Moreover, WHO advocated for first care to be provided as a continuum throughout the lifecycle including adolescence, pregnancy, childbirth, and childhood; and second in a seamless continuum that spans the home, community, and health facilities [14].

However, commonly accepted measurement for CoC in MNCH is not available. To date, CoC at the time dimension is typically presented as the collection of data on the coverage of different MNCH services from pre-pregnancy to the childhood stages [15]. Such data does not indicate how many women and children receive MNCH services continuously. An integrated indicator of CoC can highlight the gap in CoC and help to investigate how CoC improves MNCH outcomes.

### The Situation of CoC in MNCH in Ghana

Ghana is one of the SSA countries that demonstrated progress in key maternal and neonatal health indicators although it faces challenges to meet the MDG 4 and 5 targets [16–20]. Although Ghana’s under-five mortality ratio fell by 40% from 128 per 1,000 live births in 1990 to 78 per 1,000 live births in 2013, it is still globally ranked among the highest [21]. Worldwide, about 40% of under-five deaths occur within the first 28 days of live [3]. Ghana’s neonatal mortality was 29 per 1,000 live births in 2013 and accounted for 37% of all under-five deaths [21,22], maternal mortality ratio was 380 per 100,000 live births in 2013, a 50% reduction from 1990 [23].

Community-based Health Planning and Services (CHPS) initiative was regarded as a key to improving the coverage of MNCH services in Ghana [24,25], and CoC measurement would help to identify whether the improvement is continuous. CHPS was adopted in 1999, as a national health policy initiative to reduce the geographical barriers to healthcare. Initially focusing on deprived and remote areas of rural districts, CHPS endeavors to transform the primary health care system by migrating from the conventional facility-based and ‘outreach’ services to a program of mobile community-based care provided by a resident nurse [26]. CHPS scale up was motivated by the Navrongo model (an experimental study) that aimed to improve the access, efficiency, and quality of health and family planning care [26]. The principal human resources for CHPS are nurses, referred to as community health officers (CHOs). Some of them are midwives or have midwifery skills to attend emergency deliveries and make referrals should complications arise. The CHOs spend 18 months in training schools and 6 months internship for developing community liaison skills. They are provided with essential equipment and assigned to health posts, termed CHPS compounds where they live and conduct doorstep services [27,28]. CHOs are supported by community volunteers who help with community mobilization and other basic activities [28]

Despite such efforts at the community level, the coverage of delivery assisted by skilled birth attendant (SBA) and early postnatal care (PNC) is relatively low, compared with ANC. According to the Multiple Indicator Cluster Survey 2011, the coverage of delivery assisted by SBA and PNC (up to 48 hours) visit were 68.4% and 41.5%, respectively, whilst the coverage of ANC for four times or above reached 86.6% [29]. That implies that CoC at the time dimension, even if its definition was limited to the continuity from pregnancy to postpartum, was not ensured. Although various components (ANC, SBA, and PNC) of MNCH have been studied [30–34], none has assessed MNCH services as a continuum in Ghana. Whereas improvements have been made in ANC and SBA, newborn care remains a challenge although continuity in MNCH services reduces neonatal deaths risk [35,36]

This study was conducted to examine CoC completion rate and identify the gaps and factors affecting CoC completion in Ghana. [37]. The specific objectives were as follows: first, to measure the completion of CoC from the pregnancy to six weeks postpartum stages in Ghana; second, to examine the factors associated with the completion of CoC in Ghana.

## Methods

### Study setting

We collected data on background characteristics and MNCH services received by women and their infants from pregnancy to delivery, and up to six weeks after delivery. The total population of this survey area was 467,000 in 2012 [38,39]. This study was conducted as part of the formative research of the Ghana EMBRACE Implementation Research Project [37]. EMBRACE stands for Ensure Mothers and Babies Regular Access to Care; it exploits a package of evidence-based interventions to improve the health of mothers and children through the CoC approach. The Government of Japan launched EMBRACE in 2010 as a strategic initiative to accelerate efforts to help attain the health-related MDGs in developing countries, especially maternal and child health [40]. The Ghana EMBRACE Implementation Research Project was launched in 2012 and the details of the project has been published elsewhere [41]

**Fig 1:**
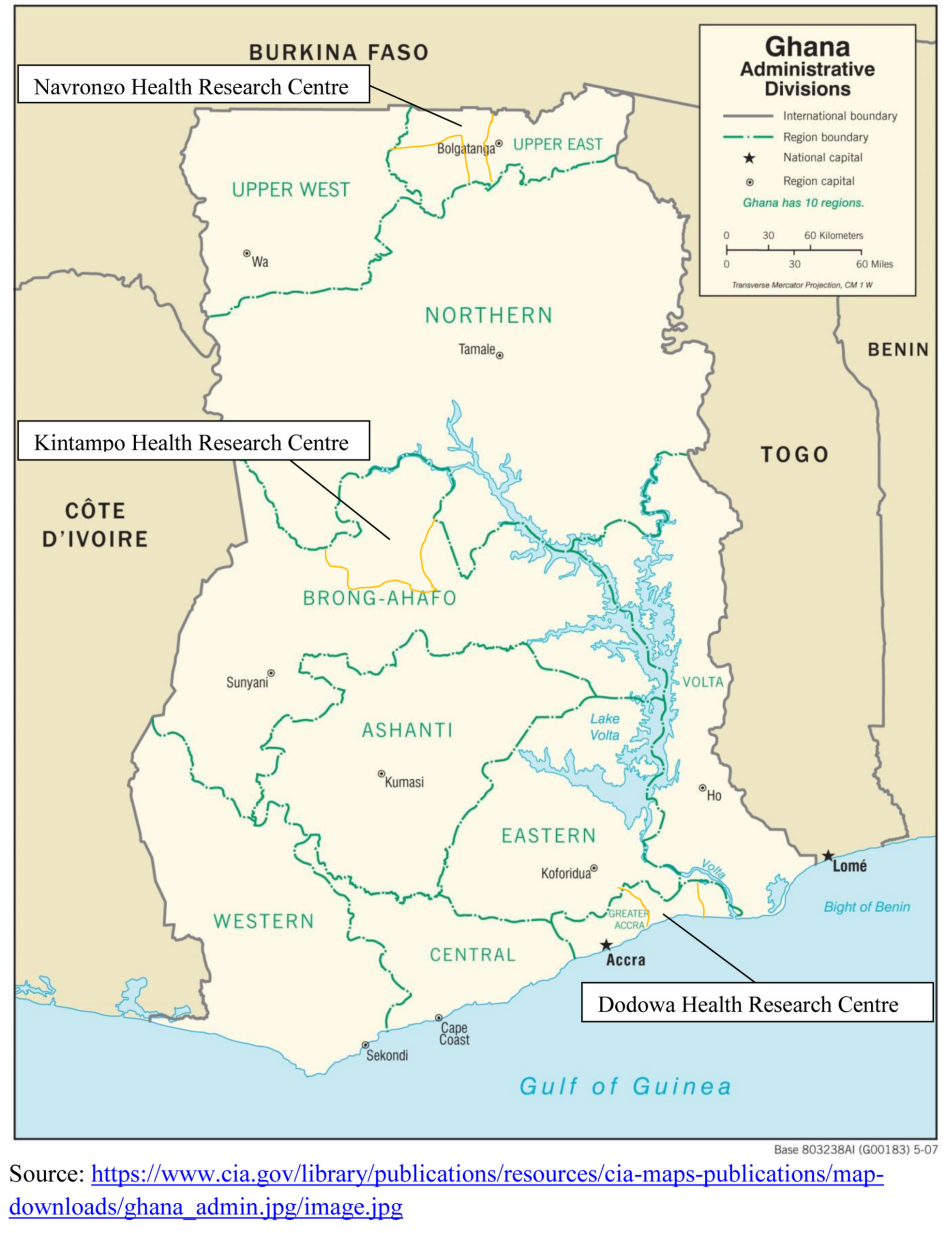
Map of Ghana showing the location of the three Health Research Centres.

This study was done in Navrongo, Kintampo, and Dodowa, where the three Health Research Centres (HRCs) of the Ghana Health Service’s (GHS) are located (Fig 1). These HRCs operate a Health and Demographic Surveillance System (HDSS). The three HRCs are strategically located to represent the three geographical belts of Ghana namely; Northern Savanah (Navrongo), Middle forest (Kintampo), and Coastal (Dodowa). The Navrongo HRC is situated in the Kassena-Nankana Districts (KNDs) of the Upper East Region of Northern Ghana. The area is predominantly rural and majority of people are subsistence farmers living in small, scattered settlements. The KNDs have one district hospital that draws from 9 health centres, 2 private clinics, and over 50 CHPS Compounds offering basic health care [42,43], manned by 7 doctors, 61 midwives, and 140 community health nurses/officers (CHNs/CHOs), CHPS started in Navrongo in 1999, and has helped to improve the health of the people in the communities. Due to the impact of CHPS, Navrongo is reported to be the only HRC on track to achieve MDG 5 in Ghana [26,38,44].

The Kintampo HRC operates within the boundaries of the Kintampo North Municipality and Kintampo South District in the Brong-Ahafo Region. The inhabitants are mainly rural, constituting about 65% of the population. The road network linking most communities to the district capital is bad, inhibiting access to health facilities. Thus, many women choose to give birth at home [45,46]. There are 7 doctors, 17 midwives and 56 CHNs/CHOs providing MNCH services in 2 hospitals supported by 6 health centres, and 34 CHPS compounds. The Dodowa HRC is located within the boundaries of the Shai-Osudoku and Ningo-Prampram districts in the Greater Accra Region, about 41 kilometers from Accra. It is fairly rural and coastal with scattered communities. The main occupations are farming, fishing and petty trading. The provision of health services is hindered by a poor road network and by the long distances between settlements [39]. There are 21 static health facilities which have 8 doctors, 34 midwives and 93 CHNS/CHOs, and 150 outreach sites mainly for public health services.

### Study design, population and sampling

This study was cross-sectional, and the target population was women aged 15 to 49 years who’s most recent pregnancy and live birth was between January 2011 and April 2013. Using a two-stage random sampling method, 1,500 pairs of women and their infants (500 from each of the three HDSS sites) were selected for interview. The primary sampling unit (PSU) was the zone or sub-district, depending on the HDSS site. The sub-districts which have populations ranging from 5,000 to 35,000 are the lowest health administration units in Ghana. There are 22 PSUs in the study area, each of which had approximately 1,200 eligible women on average for this study. Women were randomly selected from each zone or sub-district using probability proportional to size.

To calculate the sample size we assumed that the coverage of key MNCH services from pregnancy to delivery and six weeks postpartum was 15% (significance level = 0.05; power = 0.8). Data from women who had live births and children who were under six weeks old at the time of the survey were excluded. Data from women with missing information on key variables such as transportation were also excluded from the regression analyses. A total of 42 individuals with missing data on some variables (transport to delivery place, and women’s beliefs) were excluded in the final regression analysis

### Measurements

#### Dependent variable

CoC is the outcome variable in this study. The EMBRACE indicators for CoC include:

- At least four ANC visits (ANC4+) by MNCH health service providers at a health facility, in the community, or at home,
- SBA—Delivery assisted by a health professional (i.e., doctor, nurse/midwife, community health officer/nurse) at a health facility or home, and
- PNC for mother and infant within 48 hours, at two weeks, and at six weeks after delivery by MNCH health service providers at a health facility, in the community, or at home

These indicators follow the Ghana Safe Motherhood Protocol guidelines (adopted from WHO guidelines) [46,47] and the literature [9,48–50]. These CoC indicators were used to construct a binary CoC variable as follows: 1 for women who completed CoC (ANC4+, SBA, and PNC within 48 hours, at two weeks, and at six weeks), and 0 otherwise. A woman was considered to have discontinued CoC at three levels: pregnancy, delivery, and postpartum. Thus, non-achievement of ANC4+, regardless of achieving SBA and the three PNCs, was discontinuation CoC at pregnancy; non-achievement of SBA, with achievement of ANC4+ and regardless of achieving the three PNCs, was discontinuation at delivery; and a lack of any of the PNCs with achievement of ANC4+ and SBA was discontinuation at postpartum.

#### Independent variables

They were age, education, partner’s education, marital status, socio-economic status (SES), religion, location, parity, timing of pregnancy, transportation to delivery place, family support for woman (e.g. Support taking care of baby), and woman’s beliefs about childhood illnesses. Location in this study refers to geographical location, that is, the place where the three HRCs are located in Ghana as explained under study setting (Fig 1). Socio-economic status (SES) was measured using a household assets index [51]. The assets index was calculated as the first component obtained in a principal component analysis (PCA) about information on house ownership, land ownership, water source, electricity source, toilet type, and household assets including telephones, television, video decks, fridges, sewing machines, car, motorbikes, bicycles, tractors, cattle, and other livestock [52]. To measure woman’s belief about childhood illnesses, respondents were also asked whether they believe all childhood illnesses can be treated or not. In rural Ghana, some causes of new-born illnesses such as severe malaria, fontanel and “asram” (symptoms include green/black veins, a big head and newborn growing lean) are mostly believed to be spiritual and cannot be treated at the health facility [36,53,54]. Also, in rural Northern Ghana, children born with abnormalities such as hydrocephaly are sometimes believed to be spirit children (sent from the bush to destroy the family) [55], thus care is not sought for them at the health facility.

### Data collection

Through a structured questionnaire, this study collected data on CoC and most of independent variables. It also used HDSS data from three HRCs for data on ethnicity, religion, and SES. The questionnaire was developed by the Ghana EMBRACE Implementation Research Project Team in English, but the interviewers conducted interviews in local languages that the respondents spoke. The questionnaire was developed based on data collection tools used in previous studies in the same setting, including Demographic and Health Survey, Ghana Maternal Health Survey [30,56,57]. The questionnaire was pre-tested in each of the HRCs, and finalized in July 2013. Data collection using face-to-face interviews with women was undertaken from August to September 2013 with a 100% response rate.

### Statistical analysis

Descriptive analysis was performed to show the background characteristics of participants. Multivariable logistic models were performed to examine the factors associated with the CoC. The models were built using the backward selection. The final model assessed the effect of the independent variables on CoC. By using the log-likelihood test, the parsimony of the final model was checked using age “apriori” and it was not significant (LRR^2^ = 1.76, P = 0.6236). A multinomial logistic model was used to assess the factors associated with discontinuity in CoC. For the multinomial logistic regression, the dependent variable was the level of continuity in care and had four values. The base value was to receive care at ANC, delivery, and PNC. The other three values were 1) discontinued at ANC (not received ANC4+), 2) discontinued at delivery (received ANC4+ but delivered without SBA), and 3) discontinued at PNC (received ANC4+ and delivered assisted by SBA, but not received PNC three times). Stata 12 was used for analysis, and a two-sided p-value of 0.05 was considered statistically significant.

### Ethical considerations

This research obtained ethical approval from the Research Ethics Committee of the Graduate School of Medicine, the University of Tokyo; Ethics Review Committee of the Ghana Health Service; and the Institutional Review Boards of Navrongo, Kintampo, and Dodowa HRCs. Written informed consent was obtained from all participants and their confidentiality was assured.

## Results

### Background of participants

A total of 1,497 cases were included in the analysis of this study (Navrongo– 497, Kintampo– 500, and Dodowa—500). Table 1 shows that 49.9% of respondents were aged 20–29 years, followed by those aged 30–39 years (33.8%); mean age was 28.1years (SD = 6.75). Most women were married (60.8%); however, in Dodowa, cohabiting with a partner was dominant (59.4%). While 39.0% had no education, 51.3% had education at a primary or middle/junior high school level; 9.7% had secondary or higher education. Overall, 52.8% of respondents said they were Christians, although the majority (64.8%) of women in Navrongo were traditionalists. The main transport type to health facilities was on foot (49.7%, 40.8%, and 30.0% for ANC, delivery, and PNC, respectively). While women mainly travelled by commercial or private vehicles to health facilities for ANC (63.4% and 63.4%), delivery (62.6% and 45.8%) and PNC (39.8% and 45.4%) in Dodowa and Kintampo, respectively, 71.4% and 51.3% of women in Navrongo travelled on foot to the health facilities for ANC and PNC, respectively. For delivery, 35.6% and 47.3% travelled on foot and bicycle/motorbike health facilities, respectively in Navrongo.

**Table 1:** Background characteristics of the study participants (n = 1,497).

*Variable*	*Frequency (%)*			
	Navrongo, Northern Ghana	Kintampo, Mid-Ghana	Dodowa, Coastal Ghana	Overall
**Age**				
<20	45 (9.1)	39 (7.8)	46 (9.2)	130 (8.7)
20–29	268 (53.9)	227 (45.4)	252 (50.4)	747 (49.9)
30–39	148 (29.8)	185 (37.0)	173 (34.6)	506 (33.8)
40 +	36 (7.2)	36 (7.2)	25 (5.0)	97 (6.5)
Missing	0 (0.0)	13 (2.6)	4 (0.8)	17 (1.1)
**Education**				
None	148 (29.8)	244 (48.8)	192 (38.4)	584 (39.0)
Primary	139 (28.0)	96 (19.2)	109 (21.8)	344 (23.0)
Middle/JHS	138 (27.8)	138 (27.6)	148 (29.6)	424 (28.3)
Secondary	50 (10.1)	21 (4.2)	40 (8.0)	111 (7.4)
Tertiary and above	22 (4.4)	1 (0.2)	11 (2.2)	34 (2.3)
**Marital Status**				
Married	453 (91.2)	323 (64.6)	134 (26.8)	910 (60.8)
Cohabiting	8 (1.6)	90 (18.0)	297 (59.4)	395 (26.4)
Single/Divorced/Widowed	36 (7.2)	87 (17.4)	69 (13.8)	192 (12.8)
**Religion**				
Christian	99 (19.9)	231 (46.2)	460 (92.0)	790 (52.8)
Islam	5 (1.0)	174 (34.8)	30 (6.0)	209 (14.0)
Traditional	322 (64.8)	26 (5.2)	4 (0.8)	352 (23.5)
Other	27 (5.4)	42 (8.4)	6 (1.2)	75 (5.0)
Missing	44 (8.9)	27 (5.4)	0 (0.0)	71 (4.7)
**Parity**				
1	143 (28.8)	105 (21.0)	145 (29.0)	393 (26.3)
2–3	212 (42.7)	211 (42.2)	211 (42.2)	634 (42.2)
4–5	108 (21.7)	99 (19.8)	85 (17.0)	292 (19.5)
6 +	34 (6.8)	85 (17.0)	59 (11.8)	178 (11.9)
**Wanted pregnancy**				
Yes, then	328 (66.0)	331 (66.2)	216 (43.2)	875 (58.5)
Yes, but later	147 (29.6)	127 (25.4)	207 (41.4)	481 (32.1)
No, no at all	22 (4.4)	42 (8.4)	77 (15.4)	141 (9.4)
**Partner’s education level**				
None	165 (33.2)	182 (36.4)	86 (17.2)	433 (28.9)
Primary	92 (18.5)	32 (6.4)	70 (14.0)	194 (13.0)
Middle/JHS	103 (20.7)	136 (27.2)	180 (36.0)	419 (28.0)
Secondary/SSS/SHS	76 (15.3)	70 (14.0)	75 (15.0)	221 (14.8)
Tertiary and above	54 (10.9)	21 (4.2)	28 (5.6)	103 (6.9)
No partner/DK	7 (1.4)	59 (11.8)	61 (12.2)	127 (8.5)
**Wealth quintiles**				
Lowest	224 (45.0)	100 (20.0)	57 (11.4)	381 (25.5)
Second	97 (19.5)	101 (20.2)	27 (5.4)	225 (15.0)
Middle	62 (12.5)	143 (28.6)	90 (18.0)	295 (19.7)
Fourth	45 (9.1)	92 (18.4)	162 (31.4)	299 (20.0)
Highest	69 (13.9)	64 (12.8)	164 (32.8)	297 (19.8)
**Transport to ANC place**				
On foot	355 (71.4)	220 (44.0)	169 (33.8)	744 (49.7)
Bicycle/Motorking/Motorbike	118 (23.7)	3 (0.6)	3 (0.6)	124 (8.3)
Car(Taxi/Public/Private)	23 (4.6)	267 (63.4)	317 (63.4)	607 (40.6)
Missing	1 (0.2)	10 (2.0)	11 (2.2)	22 (1.5)
**Transport to delivery place**				
On foot	177 (35.6)	262 (52.4)	172 (34.4)	611 (40.8)
Bicycle/Motorking/Motorbike	235 (47.3)	8 (1.6)	4 (0.8)	247 (16.5)
Car(Taxi/Public/Private)	73 (14.7)	229 (45.8)	313 (62.6)	615 (41.1)
Missing	12 (2.4)	1 (0.2)	11 (2.2)	24 (1.6)
**Transport to PNC place**				
On foot	255 (51.3)	129 (25.8)	65(13.0)	449 (30.0)
Bicycle/Motorking/Motorbike	138 (27.8)	7 (1.4)	1 (0.2)	146 (9.8)
Car(Taxi/Public/Private)	54 (10.9)	199 (39.8)	227 (45.4)	480 (32.1)
Missing	50 (10.1)	165 (33.0)	207 (41.4)	422 (28.1)
**Woman beliefs about child illness**				
All illness can be treated	244 (49.1)	227 (45.4)	424 (84.8)	895 (59.8)
Some illness can't be treated	249 (50.1)	268 (53.6)	67 (13.4)	584 (39.8)
Missing	4 (0.8)	5 (1.0)	9(1.8)	18 (1.2)

### MNCH services coverage and CoC among women (aged 15–49) in Ghana

**Fig 2:**
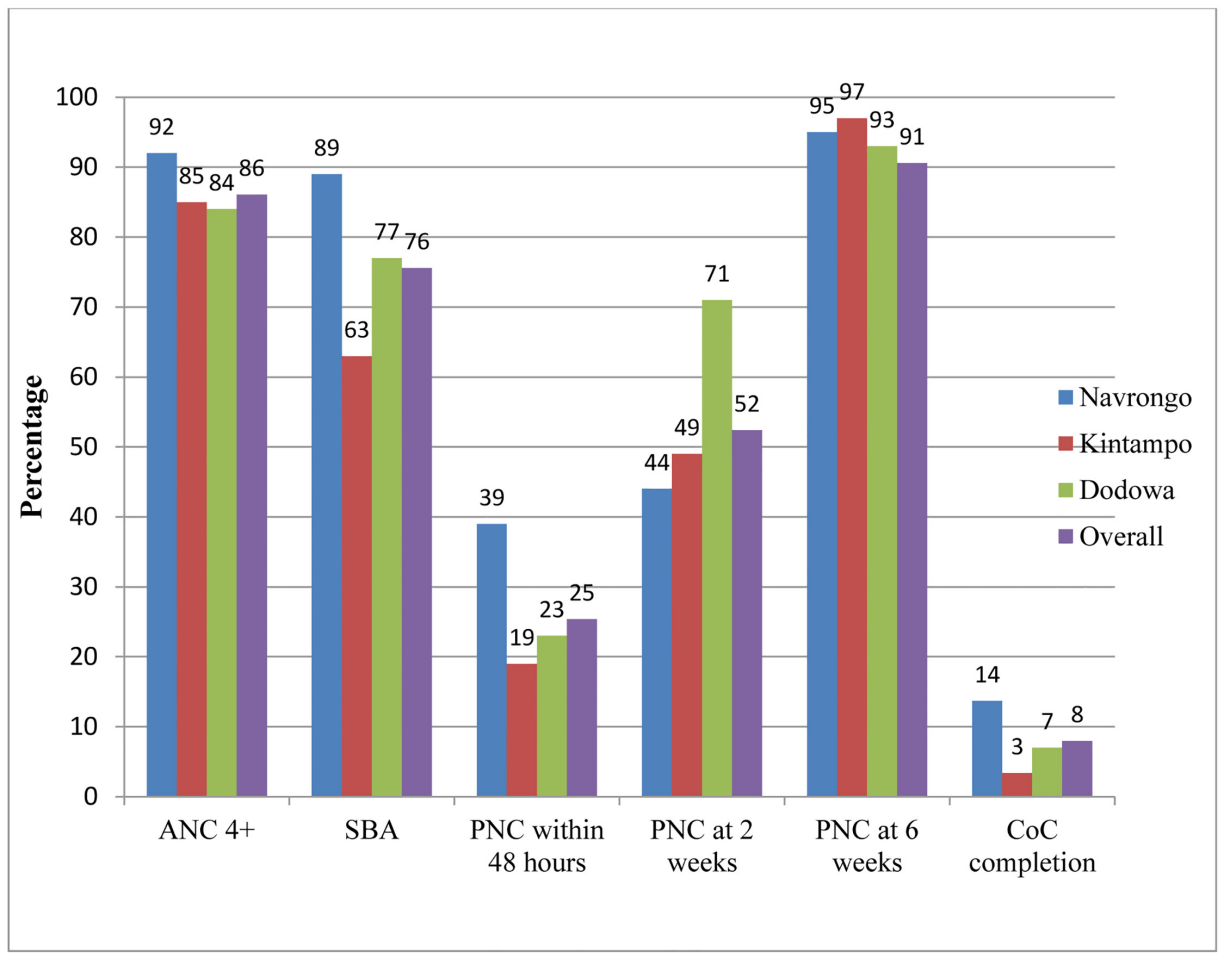
Coverage of MNCH services from pregnancy through delivery to 6 weeks postpartum in Ghana (n = 1,497).

According to Fig 2, the MNCH services coverage among all participants was 86.1% for ANC4+ and fell to 75.6% for SBA/health facility delivery; further, it drastically fell to 25.4% for PNC within 48 hours, pushed up to 52.4% for PNC at two weeks and 90.6% for PNC at six weeks after delivery. Only 8.0% of the women completed CoC, who were defined as women who received ANC4+, SBA, and PNC within 48 hours, at two weeks, and at six weeks. Women in Navrongo had the highest CoC completion rate of 14.0%, followed by Dodowa with 7.0%. The CoC completion rate for women who attained ANC4+ and had SBA/health facility delivery, but lacked either of three PNCs was 60.0%. Women who attained ANC4+, had no SBA, and defaulted in one of the three PNCs was 16.0%.

### Factors associated with CoC completion in Ghana

The unadjusted analysis demonstrated an association between location and CoC completion (Table 2). Compared to Navrongo, women in Kintampo and Dodowa were 78% (95% CI 0.12–0.39) and 52% (95% CI 0.31–0.73) less likely to complete CoC, respectively. The association between geographical location and CoC completion remained significant after adjusting for some confounders in the model for women in Kintampo (aOR = 0.36; 95% CI 0.15–0.89).

**Table 2:** Multivariable logistic regression of factors associated with CoC completion in Ghana (n = 1,417).

Variables	Univariate		Multivariate	
	OR	95%CI	aOR	95%CI
**Location**				
Navrongo	1		1	
Kintampo	0.22	(0.12–0.39)***	0.36	(0.15–0.89)*
Dodowa	0.48	(0.31–0.73)**	0.61	(0.25–1.48)
**Age**				
15–19	1		1	
20–29	1.19	(0.57–2.45)	1.22	(0.54–2.75)
30–39	1.15	(0.54–2.42)	1.03	(0.43–2.48)
40–49	0.88	(0.30–2.56)	0.75	(0.23–2.48)
**Education**				
None	1		1	
Primary	0.80	(0.50–1.39)	0.56	(0.30–1.05)
Middle	1.39	(0.89–2.18)	1.06	(0.61–1.87)
Secondary	1.28	(0.62–2.63)	0.61	(0.25–1.50)
Tertiary and above	3.35	(1.38–8.14)**	0.86	(0.26–2.87)
**Marital status**				
Married	1		1	
Cohabiting	0.45	(0.27–0.75)**	0.56	(0.28–1.12)
Single/Divorced/Widowed	0.44	(0.22–0.89)*	0.62	(0.27–1.43)
**Religion**				
Christian	1		1	
Islam	0.59	(0.27–1.26)	0.91	(0.36–2.29)
Traditional	2.44	(1.62–3.70)***	2.44	(1.12–5.28)*
Other	1.32	(0.69–2.55)	1.31	(0.52–3.26)
**Parity**				
1	1			
2–3	0.74	(0.47–1.17)		
4–5	1.05	(0.62–1.76)		
6 -	0.71	(0.36–1.41)		
**Wanted pregnancy**				
Yes, then	1		1	
Yes, but later	1.56	(1.06–2.29)*	1.75	(1.11–2.76)*
No, not at all	0.47	(0.18–1.20)	0.63	(0.21–1.88)
**Partner’s education**				
None	1		1	
Primary	0.80	(0.40–1.58)	0.64	(0.30–1.35)
Middle	0.97	(0.58–1.61)	1.05	(0.56–1.97)
Secondary	1.01	(0.55–1.85)	0.94	(0.44–2.00)
Tertiary and above	2.74	(1.48–5.04)***	2.77	(1.17–6.55)*
Don’t know/NA	0.81	(0.37–1.81)	1.27	(0.47–3.44)
**Wealth quintiles**				
Highest	1		1	
Fourth	0.46	(0.24–0.88)*	0.55	(0.27–1.11)
Middle	0.62	(0.35–1.12)	0.78	(0.38–1.60)
Second	0.78	(0.46–1.35)	0.66	(0.32–1.38)
Lowest	0.96	(0.56–1.65)	0.69	(0.31–1.55)
**Transport to ANC place**				
On foot	1		1	
Bicycle/Motorking/Motorbike	1.30	(0.72–2.36)	0.72	(0.36–1.44)
Car(Taxi/Public/Private)	0.56	(0.37–0.86)**	0.87	(0.49–1.55)
**Transport to delivery place**				
On foot	1		1	
Bicycle/Motorking/Motorbike	2.80	(1.70–4.61)***	1.56	(0.87–2.80)
Car(Taxi/Public/Private)	1.54	(0.98–2.41)	2.05	(1.16–3.61)*
**Transport to PNC place**				
On foot	1		1	
Bicycle/Motorking/Motorbike	1.40	(0.80–2.43)	0.72	(0.80–2.43)
Car(Taxi/Public/Private)	1.01	(0.66–1.51)	0.87	(0.66–1.51)
**Woman beliefs about child illness**				
All illness can be treated	1		1	
Some illness can't be treated	0.49	(0.32–0.74)***	0.37	(0.23–0.61)***
**Family help take care of baby**				
Yes	1		1	
No	1.61	(1.07–2.43)*	1.08	(0.67–1.73)
**Family accompany woman to health facilities**				
Yes	1			
No	0.80	(0.55–1.16]		
**Family encourage woman to seek care**				
Yes	1		1	
No	1.61	(1.07–2.42)*	1.49	(0.95–2.33)

Note:

*p< 0.05

**p< 0.01

***p< 0.001

Besides geographical location, various individual-level factors were associated with CoC completion in the adjusted analysis (Table 2).Women whose partners had tertiary education were about three times more likely to complete CoC (aOR = 2.77; 95% CI 1.17–6.55). Compared to Christian women, the chance of completing CoC by traditional women was over two times better (aOR = 2.44; CI 1.12–5.28). Women who believed some childhood sicknesses cannot be treated were about 60% less likely to complete CoC (95% CI 0.23–0.61).Women who said that they did not want the pregnancy at the time but later were about twice more likely to complete CoC (aOR = 1.75; 95% CI 1.11–2.76) compared to those who said they wanted the pregnancy at the time. Women who used a car to the health facility for delivery were about twice more likely to complete CoC (aOR = 2.05; 95% CI 1.16–3.61).

### Factors associated with CoC completion at specific HDSS sites in Ghana

Analysis was carried out to identify specific factors associated with CoC completion rate in each of the three HRCs (Table 3). Ethnicity, marital status, timing of pregnancy, and beliefs about childhood illnesses were associated with CoC completion in Navrongo. At Kintampo, the only factor that demonstrated a significant association with CoC completion was the type of transport used to travel to the place of delivery (aOR = 4.77; 95% CI 1.39–16.43). Compared to women who travelled by foot to the delivery place, those who went by vehicle were nearly five times more likely to complete CoC. At Dodowa, a woman’s marital status (aOR = 0.79; 95% CI 0.34–1.88), and type of transport to the delivery place (aOR = 2.93; 95% CI 1.04–8.25) were the factors that reached significant association levels with CoC completion.

**Table 3:** Multivariable logistic regression models of factors associated with CoC completion (by location) in Ghana.

Variables	Adjusted Odds Ratios (95% CI)					
	Model I		Model II		Model III	
	Navrongo (Northern Ghana)		Kintampo(Mid-Ghana)		Dodowa (Coastal Ghana)	
**Age**						
15–19	1		1		1	
20–29	0.58	(0.17–1.87)	0.42	(0.09–1.95)	1.57	(0.28–8.85)
30–39	0.44	(0.12–1.60)	0.22	(0.04–1.17)	1.18	(0.19–7.27)
40–49	0.40	(0.07–2.24)	0.23	(0.08–2.80)	0.68	(0.05–9.93)
**Education**						
None	1		1		1	
Primary	0.49	(0.19–1.27)	0.40	(0.09–1.90)	0.80	(0.22–2.87)
Middle	1.03	(0.41–2.59)	0.37	(0.08–1.65)	1.20	(0.42–3.49)
Secondary and above	0.77	(0.19–3.05)	1.01	(0.21–2.68)	0.90	(0.20–3.90)
**Marital status**						
Married	1		1		1	
Cohabiting	NA		1.47	(0.37–5.91)	0.79	(0.34–1.88) *
Single/Divorced/Widowed	0.13	(0.02–0.87)*	0.51	(0.08–3.11)	1.23	(0.34–4.51)
**Ethnicity**						
Kassena	1					
Nankana	3.00	(1.35–6.67)**				
Other	0.21	(0.03–1.78)				
**Wanted pregnancy**						
Yes, then	1		1		1	
Yes, but later	2.50	(1.15–5.47)*	0.82	(0.20–3.45)	1.62	(0.70–3.73)
No, not at all	0.48	(0.05–5.00)	1.83	(0.40–8.35)	0.23	(0.03–2.05)
**Partner’s education**						
None	1		1		1	
Primary	0.89	(0.32–2.44)	1.02	(0.38–4.79)	0.85	(0.17–4.24)
Middle	0.94	(0.36–2.51)	2.06	(0.43–9.91)	0.65	(0.18–2.34)
Secondary	1.16	(0.36–3.72)	1.47	(0.21–10.33)	0.79	(0.19–3.40)
Tertiary and above	2.72	(0.70–10.47)	2.59	(0.21–31.9)	2.25	(0.45–11.18)
Don’t know/NA	NA		3.05	(0.69–13.62)	0.71	(0.14–3.61)
**Wealth quintiles**						
Highest	1					
Fourth	0.90	(0.31–2.57)	0.53	(0.09–3.20)	1.77	(0.67–4.66)
Middle	0.63	(0.20–1.94)	2.44	(0.56–10.62)	1.00	(0.33–3.08)
Second	1.14	(0.37–3.52)	1.08	(0.20–5.79)	0.29	(0.06–1.52)
Lowest	0.51	(0.15–1.71)	0.72	(0.11–4.63)	1.00	(0.33–3.08)
**Woman beliefs about child illness**						
All illness can be treated	1		1			
Some illness can't be treated	0.38	(0.19–0.76)**	1.1	(0.37–3.23)		
**Transport to delivery place**						
On foot	1		1		1	
Bicycle/Motorking/Motorbike	0.89	(0.40–1.95)	NA		NA	
Car(Taxi/Public/Private)	0.33	(0.08–1.38)	4.77	(1.39–16.43)**	2.93	(1.04–8.25)*
**Transport to PNC place**						
On foot	1					
Bicycle/Motorking/Motorbike	1.38	(0.53–3.18)				
Car(Taxi/Public/Private)	3.75	(0.97–14.47)				
**Family help take care of baby**						
Yes	1		1		1	
No	1.47	(0.65–3.36)	0.63	(0.20–1.98)	1.26	(0.63–3.16)
**Family encourage mother to seek care**						
Yes	1		1		1	
No	1.04	(0.52–2.07)	1.62	(0.56–4.67)	1.83	(0.67–5.01)

Note:

*p< 0.05

**p< 0.01

### Risk factors associated with discontinuity in CoC completion at the three stages of care (pregnancy, delivery, and postpartum)

Relative to Navrongo, women in Kintampo and Dodowa were at a significantly higher risk of discontinuing CoC at pregnancy and delivery (Table 4). For instance, women in Kintampo were at a four times higher risk of discontinuing CoC at pregnancy (RR = 4.28; 95%CI 1.39–13.15) and 16-fold increased risk of discontinuity at delivery (RR = 15.89; 95%CI 5.36–47.07). However, women at Dodowa only had a higher risk of discontinuing CoC at delivery (RR = 7.63; 95%CI 2.40–22.20).

**Table 4:** Multinomial logistic model of the factors associated with discontinuity in CoC in Ghana.

Variable	Discontinued at ANC		Discontinued at delivery		Discontinued at PNC	
	RR	95% CI	RR	95% CI	RR	95% CI
**Location**						
Navrongo	1		1		1	
Kintampo	4.28	(1.39–13.15)**	15.89	(5.36–47.07)***	1.99	(0.83–4.80)
Dodowa	2.80	(0.85–9.20)	7.63	(2.41–24.22)**	1.32	(0.56–3.10)
***Age***						
15–19	1		1		1	
20–29	0.97	(0.38–2.50)	0.97	(0.38–2.45)	0.88	(0.39–2.00)
30–39	1.01	(0.36–2.82)	0.95	(0.35–2.58)	1.13	(0.47–2.71)
40–49	1.47	(0.37–5.83)	0.65	(0.16–2.59)	1.54	(0.47–5.07)
***Education***						
None	1		1		1	
Primary	1.99	(0.95–4.13)	1.81	(0.89–3.68)	1.76	(0.93–3.33)
Middle	0.68	(0.34–1.37)	0.54	(0.28–1.06)	1.00	(0.57–1.75)
Secondary	0.70	(0.19–2.59)	0.63	(0.18–2.21)	1.71	(0.70–4.18)
Tertiary and above	N/A		0.57	(0.05–6.06)	1.09	(0.33–3.55)
**Marital status**						
Married	1		1		1	
Cohabitating	2.95	(1.29–6.74)**	1.74	(0.79–3.83)	1.56	(0.77–3.16)
Single/Divorced/Widowed	3.41	(1.32–8.83)*	1.60	(0.63–4.10)	1.57	(0.68–3.62)
***Religion***						
Christian	1		1		1	
Islam	1.43	(0.50–4.04)	1.16	(0.43–3.16)	1.01	(0.40–2.55)
Traditional	0.77	(0.27–2.23)	1.33	(0.49–3.62)	0.41	(0.19–0.91)*
Others	1.00	(0.33–3.05)	0.83	(0.29–2.40)	0.82	(0.33–2.07)
***Wanted pregnancy***						
Yes, then	1		1		1	
Yes, but later	1.00	(0.58–1.75)	0.79	(0.46–1.34)	0.55	(0.35–0.86)*
No	2.03	(0.62–6.65)	2.18	(0.68–6.98)	1.47	(0.49–4.40)
**Partner’s education**						
None	1		1		1	
Primary	1.14	(0.47–2.75)	1.29	(0.55–3.03)	1.66	(0.78–3.55)
Middle	0.53	(0.25–1.14)	0.82	(0.40–1.69)	1.05	(0.56–1.98)
Secondary	0.70	(0.27–1.76)	0.89	(0.36–2.17)	1.12	(0.52–2.39)
Tertiary and above	0.13	(0.03–0.57)**	0.24	(0.07–0.84)*	0.43	(0.18–1.01)
Don’t know/NA	0.49	(0.16–1.50)	0.47	(0.16–1.41)	0.91	(0.33–2.48)
**Wealth Quintiles**						
Highest	1		1		1	
Fourth	2.41	(0.99–5.85)	3.28	(1.33–8.10)**	1.78	(0.87–3.62)
Middle	2.61	(1.08–6.31)*	3.59	(1.47–8.78)**	1.08	(0.53–2.22)
Second	2.46	(0.96–6.26)	6.17	(2.47–15.42)***	1.25	(0.60–2.62)
Lowest	2.63	(0.95–7.24)	7.07	(2.62–19.13)***	1.34	(0.59–3.03)
***Transportation to ANC place***						
On foot	1		1		1	
Bicycle/Motorking/Motorbike	1.72	(0.65–4.60)	0.78	(0.28–2.23)	1.24	(0.62–2.48)
Car(Taxi/Public/Private)	1.74	(0.89–3.37)	1.35	(0.72–2.54)	0.85	(0.48–1.50)
***Woman beliefs about child illness***						
All illness can be treated	1		1		1	
Some illness can't be treated	3.25	(1.80–5.88)***	2.66	(1.51–4.68)***	2.57	(1.56–4.23)***
***Family take care of baby for mother***						
Yes	1		1		1	
No	0.81	(0.46–1.41)	0.84	(0.49–1.45)	0.98	(0.61–1.57)
***Family encourage mother to seek care***						
Yes	1		1		1	
No	0.75	(0.43–1.29)	0.58	(0.35–0.97)	0.69	(0.44–1.08)

Note:

*p< 0.05

**p< 0.01

***p< 0.001

N/A–“Not applicable” indicating data not available for specific category

Marital status, partner’s education, wealth quintiles, and woman’s beliefs about child illness were other factors significantly associated with a higher risk of discontinuing CoC at the pregnancy and delivery stages (Table 4). Compared to the married, cohabiting or single women had a three times higher risk of discounting CoC at pregnancy (RR = 2.95; 95%CI 1.29–6.74 for cohabitating, and RR = 3.41; 95%CI 1.32–8.83 for single). Low SES showed a higher risk of discontinuity of CoC at both ANC and delivery. Partners who had a tertiary education were associated with a lower risk of discontinuity of CoC at ANC (RR = 0.13; 95% CI 0.03–0.57), and delivery (RR = 0.24; 95% CI 0.07–0.84). Women who believed that some childhood sicknesses cannot be treated were three times more likely to discontinue CoC at ANC (RR = 3.25; 95%CI 1.80–5.88) and delivery (RR = 2.66; 95%CI 1.51–4.68).

## Discussion

This study revealed that most of the women and their children did not receive MNCH services continuously across the pregnancy, delivery, and postpartum stages. Only 8% of women completed CoC in the study site. The factors that significantly influenced low CoC completion rate were location, marital status, education, SES, pregnancy timing, transport to delivery place and cultural beliefs.

Although previous studies in Ghana [58–62] and elsewhere [63–66] found similar factors associated with MNCH service use, this study is unique as it had a different outcome variable, or CoC completion [31,32,67]. The outcome variable captured the aspect of CoC in MNCH, which was not addressed in these other studies. Nevertheless, factors associated with the outcome variable was similar in this study to such previous studies [31,33,34]

The study findings demonstrated differences in CoC completion rates ranging from 14% in Navrongo to 3% in Kintampo. It may be mainly attributable to difference in the development of CHPS at different phases across locations [24]. CHPS is widespread in Navrongo than Kintampo and Dodowa [68], and thus CoC completion was higher in Navrongo.

This study identified the low coverage of PNC within the first 48 hours postpartum as a major gap in CoC. Such low coverage of PNC has been observed in SSA [49,50,69]. The postpartum period (first six weeks after birth), particularly the first 48 hours, is critical to the health and survival of the mother and her newborn [70]. A lack of skilled care during this period may lead to death or diseases and missed opportunities for essential health education [71]. Our findings of 25% coverage of PNC within 48 hours after delivery was much lower than an earlier reported coverage of 68% which applied a different definition of the coverage [72,73]. Other studies [56] considered only the woman who received care from a health professional within 48 hours either before or after discharging from a health facility. That is, a woman was regarded as received PNC within 48 hours if she received check-up and discharge from a health facility soon after delivery. This study considered both woman and newborn who received care before and after discharge for the cases of such an early discharge. Also, the findings showed that Ghana’s recent improvement in SBA [71,74,75] has not resulted in high PNC coverage within 48 hours.

Two factors may account for the low PNC coverage within 48 hours after delivery. First, the early discharge (less than 24hours) of women who delivered at health facilities without receiving the first PNC [50,76]; and second, the inability of either community health workers to visit women who delivered at home or the inability of women who delivered at home to visit health facilities for PNC within 48 hours.

In this study, a woman’s worldview affected her local understandings of disease etiology and the use of MNCH services from pregnancy to postpartum. For instance, seeking of health care may be delayed in situations in which a woman perceives the cause of a health problem as spiritual rather than physical [77–79]. Consistent with literature in similar settings [77–80], the findings revealed that women were less likely to complete CoC if they held a belief that some childhood illnesses cannot be treated. Although the association between a woman’s belief about childhood illness and CoC completion was significant in the overall regression model, it remained significant only for women in Navrongo in the stratified analysis by location. This is in line with the findings of Moyer and colleagues in a recent study [77] on how social factors influence facility-based delivery in rural Northern Ghana.

The findings demonstrated that partner’s education was positively associated with CoC completion. This is consistent with previous findings in Ghana [61,62,72,81] and Kenya [82]. In some parts of Ghana where the patriarchal system is practiced, men play a significant role in the decision-making process in their families. Thus, men’s education could be an important strategy for improving MNCH in the country. The finding that women’s pregnancy intentions were associated with CoC completion is consistent with the literature [83–86]. The results also indicated that women who wanted their pregnancy later were likely to complete CoC, and remained significant only among women in Navrongo in the stratified analysis. This contrasts with a study in Tanzania by Exavery et al [87], which showed that mistimed and unwanted pregnancies are associated with late initiation of ANC. The reason why mistimed pregnant women were more likely to complete CoC is not clear in this study. Further studies are needed to unearth the reasons for this.

In contrast with other findings [61,81,86], the association between SES and CoC completion was not significant in logistic regression modelling. Interestingly, the multinomial regression model showed that poorer women have a higher risk of discontinuing CoC at ANC and delivery, and this is consistent with the literature [58,61,88,89]. This suggests that women’s SES may not be contributing to the interruption of CoC at the postpartum stage once they received care up to the delivery stage.

A strong association was detected between type of transport used to health facility and CoC completion. Geographical distance and travel time to health facilities are known to play a major role in women’s use of MNCH service in Ghana [62,90] and elsewhere [80,91,92]. Similar to other research findings in Ghana [62,89], Tanzania[80] and China [92], the results revealed that women who had access to good transport (private car/ambulance) to the delivery place were over twice more likely to complete CoC compared to those who travel on foot. In most rural areas, commercial transport is not regular; but only available on market days. Therefore, travelling to health facilities for maternal care is particularly onerous and expensive to rural women on non-markets days and at night [80,92]. Of interest is the recent work of Rishworth [89] in a study on women’s use of MNCH services in Northern Ghana and Esena and Mary [61] on factors associated with skilled delivery in Coastal Ghana, found a lack of available transport to be a barrier.

This study highlighted differences in the factors associated with CoC completion by geographic location. Transport to the delivery place was a major factor associated with CoC completion in Kintampo and Dodowa, but not in Navrongo, which could be attributed to the development of CHPS with more health facilities and midwives in rural areas [24,26,68] in Navrongo. A recent study by Naariyong et al [93] showed that women in CHPS areas received better quality ANC than their colleagues in non-CHPS communities. Compared to the other sites (Kintampo and Dodowa), Navrongo have the highest number of CHPS compounds delivering health care in rural and hard to reach communities. Most of the CHPS compounds in Navrongo now have resident midwives providing MNCH services (ANC, SBA, PNC), thus, removing geographical access barrier. Therefore, Kintampo and Dodowa need more CHPS compounds. Also, the result indicated that cohabitation was a major barrier to CoC completion in Dodowa than in Kintampo and Navrongo. In this study, approximately 60% of women in Dodowa were cohabitating. Cohabiting women had a higher risk of discontinuing CoC right from ANC [84]. Studies have shown that unmarried women were more associated with an unintended pregnancy resulting in late initiation of ANC [83].

One of the major limitations of this study is that the main components of MNCH CoC services were measured based on women’s recall response, which is prone to recall bias. Despite this, we believe it is the best method to collect information on MNCH service seeking behavior by women from pregnancy, to delivery, and postnatal care. Also, we did not have data on the location of respondents (rural/urban) and distance to a health facility. However, this study overcame this by controlling for the difference in location across districts/HRCs in the analysis. Moreover, since this is a cross sectional study, it did not establish causality. Finally, we did not include service availability and other adjustable program factors, which may influence utilization of MNCH services such as demand creation efforts including home visits by CHOs.

## Conclusions

This study presented the results of an integrated measure of CoC from the pregnancy to postpartum stages and showed that CoC completion rate was less than 10% among women. The greatest gap and contributor to the low in CoC completion was between delivery and PNC within 48hours after delivery. Factors associated with low CoC completion rate, include HRC geographical location, marital status, religion, education, transportation to delivery place, and cultural beliefs. Such factors were different across geographical locations.

These study findings call for attention to interventions aimed at increasing PNC for women and their infants within 48hours after delivery, thereby increasing CoC completion rate. Such interventions should be tailored towards the needs of women in Navrongo, Dodowa and Kintampo to appropriately address the factors hindering CoC completion.
